# Effects of NDRG1 family proteins on photoreceptor outer segment morphology in zebrafish

**DOI:** 10.1038/srep36590

**Published:** 2016-11-04

**Authors:** Shimpei Takita, Yasutaka Wada, Satoru Kawamura

**Affiliations:** 1 Department of Biological Sciences, Graduate School of Science, Osaka University, Yamada-oka 1-3, Suita, Osaka, 565-0871, Japan; 2 Graduate School of Frontier Biosciences, Osaka University, Yamada-oka 1-3, Suita, Osaka, 565-0871, Japan

## Abstract

Rods and cones are functionally and morphologically distinct. We previously identified N-myc downstream-regulated gene 1b (*ndrg1b*) in carp as a cone-specific gene. Here, we show that NDRG1b and its paralog, NDRG1a-1, contribute to photoreceptor outer segment (OS) formation in zebrafish. In adult zebrafish photoreceptors, NDRG1a-1 was localized in the entire cone plasma membranes, and also in rod plasma membranes except its OS. NDRG1b was expressed specifically in cones in the entire plasma membranes. In a developing retina, NDRG1a-1 was expressed in the photoreceptor layer, and NDRG1b in the photoreceptor layer plus inner nuclear layer. Based on our primary knockdown study suggesting that both proteins are involved in normal rod and cone OS development, NDRG1a-1 was overexpressed or NDRG1b was ectopically expressed in rods. These forced-expression studies in the transgenic fish confirmed the effect of these proteins on the OS morphology: rod OS morphology changed from cylindrical to tapered shape. These taper-shaped rod OSs were not stained with *N,N’*-didansyl cystine that effectively labels infolded membrane structure of cone OS. The result shows that rod OS membrane structure is preserved in these taper-shaped OSs and therefore, suggests that tapered OS morphology is not related to the infolded membrane structure in cone OS.

In the vertebrate retina, there are two types of photoreceptors, rods and cones. They are distinct in function and morphology^1^,^2^. Rods are more sensitive to light than cones, so that rods govern scotopic vision and cones mediate photopic vision including color discrimination. In addition, briefer responses allow cones to detect light stimuli with higher time resolution. The structure of the outer segment (OS) is also remarkably different from each other. Rod OS is macroscopically cylindrical and consists of a stack of disk membranes surrounded by plasma membrane. Cone OS is macroscopically conical or tapered, and consists of topologically continuous infolded plasma membrane.

In our previous study using purified carp rods and cones^3^, we found genes specifically expressed in cones including N-myc downstream-regulated gene-like (formerly *ndrg1l,* now *ndrg1b*). NDRG protein family consists of four protein subtypes; NDRG1, NDRG2, NDRG3 and NDRG4^4^. Among them, *NDRG1* mRNA is ubiquitously expressed in most human tissues, but its expression in the mammalian retina has not been studied. NDRG1 protein seems to be involved in many biological functions in an organ- or tissue-specific manner^5^. For example, NDRG1 protein is identified as a metastasis suppressor in a variety of cancers^5^,^6^. In neurons, *NDRG1* gene is identified as a responsible gene for Charcot-Marie-Tooth disease type 4D which is characterized by Schwann-cell dysfunction of peripheral nervous system^7^. Recent study showed that the lack of this gene induces demyelination in mice, a phenocopy of this human disease^8^,^9^. However, functional roles of NDRG1 proteins or their functional mechanisms have not been clearly understood. It is partly because studies made under *in vitro* conditions do not always agree with those made under *in vivo* conditions^10^.

In the present study, we made efforts to understand the role of NDRG1 family proteins in zebrafish retina. In teleost, there are two homologs of *ndrg1* gene, *ndrg1a* (consisting of two variants, *ndrg1a-1* and *ndrg1a-2*) and *ndrg1b*. Zebrafish *ndrg1a* gene is known to be expressed ubiquitously including in the eye (http://zfin.org/action/figure/all-figure-view/ZDB-PUB-040907-1?probeZdbID=ZDB-CDNA-040425-2588)^11^. However, it is not known whether *ndrg1a* is *ndrg1a-1* and/or *ndrg1a-2*, because two splice variants, *ndrgia-1* and *ndrg1a-2*, were not distinguished in that study. Zebrafish *ndrg1b* gene is preferentially expressed in the eye (http://zfin.org/action/figure/all-figure-view/ZDB-PUB-040907-1?probeZdbID=ZDB-CDNA-040425–2588)^11^. In our present study, we cloned mRNAs of zebrafish NDRG1 family proteins, *ndrg1a-1*, *ndrg1a-2* and *ndrg1b*, and examined their protein localization in zebrafish rods and cones. Our study using morpholinos seemed to suggest involvement of *ndrg1a-1* and *ndrg1b* in normal development of rods and cones. Therefore, *ndrg1a-1*, which is expressed in both zebrafish rods and cones, was overexpressed in rods, and *ndrg1b*, which is specifically expressed in zebrafish cones similarly as in carp, was ectopically expressed in zebrafish rods. Our results suggest that NDRG1 family proteins are involved in forming a normal OS shape in zebrafish photoreceptors.

## Results

### Identification of three NDRG1 family proteins in zebrafish

In our previous study in carp^3^, a partial cDNA sequence (*ndrg1l*) possibly encoding NDRG1b protein was found as a gene specifically expressed in cones. To ascertain that putative carp *ndrg1l* is really a carp ortholog of zebrafish *ndrg1b*, full-length cDNA clones containing the partial sequence found previously were isolated from a carp retinal cDNA library and verified by DNA sequencing (DDBJ accession number, LC102483.1).

The deduced amino acid sequence identity is 88% between carp NDRG1L (hereafter, we call it carp NDRG1b) and zebrafish NDRG1b (Supplementary Fig. S1a). Phylogenetic tree was constructed using determined carp NDRG1b amino acid sequence and those of other NDRG family proteins in the data base including NDRG1, NDRG2, NDRG3 and NDRG4 in other animals (Supplementary Fig. S1b). All of these results supported the notion that carp NDRG1L was the ortholog of zebrafish NDRG1b, which in turn suggests that zebrafish NDRG1b is also specifically expressed in zebrafish cones.

Therefore, we switched the experimental animal from carp to zebrafish, because genetic study is more practical in zebrafish than in carp. In zebrafish, two splice variants are known to be present for *ndrg1a* gene: *ndrg1a-1* and *ndrg1a-2.* They are different only at their N-terminal amino acid sequences, Met1-Ala7 in NDRG1a-1 and Met1-Lys30 in NDRG1a-2 (Supplementary Fig. S1c). We cloned all of these three genes, *ndrg1a-1*, *ndrg1a-2* and *ndrg1b*, in zebrafish. There were many single nucleotide polymorphisms in these homologs (for further information about the substitutions, see Supplementary Methods). As the representative of each NDRG1 family protein for functional analysis hereafter, we selected the variant that shows minimum amino acid substitution compared with the amino acid sequence of a zebrafish NDRG1 family protein reported in the database (NP_001121825.1 for NDRG1a-1, NP_998513.2 for NDRG1a-2 and NP_956986.1 for NDRG1b). The representatives in this study were NDRG1a-1_1 (LC093848.1), NDRG1a-2_1 (LC093852.1) and NDRG1b_1 (LC093857.1). As for NDRG1a-1_1 and NDRG1a-2_1, amino acid sequences were the same as those in the database. There were 4 amino acid substitutions in NDRG1b_1: Cys was substituted for Arg (at position 39), Gly for Ser (142), Leu for Met (148) and Ser for Asn (200).

### Subcellular localization of NDRG1 family proteins in adult zebrafish rods and cones

**Figure 1:**
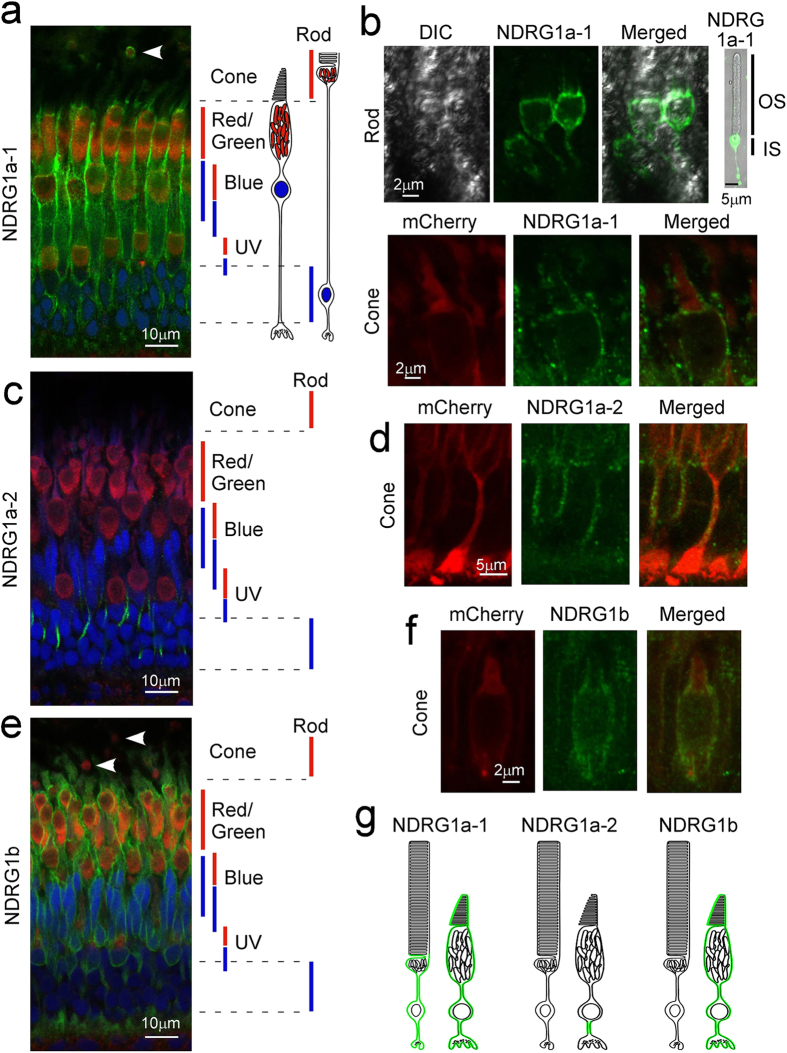
Subcellular localization of NDRG1 family proteins in zebrafish photoreceptors. Adult zebrafish retinas were immunostained with specific anti-NDRG1a-1 (**a**,**b)**, anti-NDRG1a-2 (**c**,**d**) or anti-NDRG1b (**e**,**f**) antiserum (all with green signals). Mitochondria and nucleus were counterstained with anti-Tom20 antibody (red signals) and Hoechst 33342 (blue signals), respectively (**a**,**c**,**e**). In **a**, **c** and **e**, approximate positions of ellipsoid containing mitochondria (red bars) and nucleus (blue bars) for each cone type (red/green-, blue- and UV-sensitive cones) and for rods are indicated. NDRG1a-1 was found to be expressed in the ellipsoid but not in the OS in rods (*arrowhead* in **a,** and upper panels in **b**). NDRG1a-1 was also found both in the OS and ellipsoid in cones (**a** and lower panels in **b**). NDRG1a-2 was found in the thin process of a cone (**c**,**d**). NDRG1b was found in the entire region of a cone (**e**,**f**) but not in rod ellipsoid (*arrowheads* in **e**). In **a**, **c** and **e**, wildtype zebrafish retinas were used, and in the detailed study in **b**, **d** and **f**, the retinas consisting of cones expressing mCherry-HrasCAAX were used to readily identify cones. (**g**) Schematic representation of localization of NDRG1 family proteins in adult zebrafish photoreceptors (shown in green). DIC, differential interference contrast image.

To examine the localization of NDRG1 family proteins in zebrafish retina, NDRG1a-1 and NDRG1a-2 partial peptides and NDRG1b whole protein (Supplementary Fig. S1c) were used to raise antiserum and each antiserum was purified (see Supplementary Methods). With specific antiserum against an NDRG1 family protein (Supplementary Fig. S1d, see also Supplementary Methods), localization of each protein was immunoprobed in adult zebrafish in the eye (Supplementary Fig. S2) and at the photoreceptor layer (Fig. 1). The results revealed that NDRG1a-1 is expressed specifically in the photoreceptor layer (Supplementary Fig. S2a), and that at the photoreceptor layer, it is expressed in rods plus all four types of cones (green signals in Fig. 1a,b; positions of the ellipsoid and the nucleus are indicated by vertical red and blue bars, respectively, in rods and all types of cones in Fig. 1a). In rods, entire cell membranes other than those of OS seem to be immunopositive (green signals in Fig. 1a and in upper panels in Fig. 1b; see also green signal surrounding red mitochondrial signal indicated by an arrowhead in Fig. 1a). At the basal part of rod OS, immunopositive thin processes are extending from the inner segment to the OS (Fig. 1b, upper panels), and they could be the calycal processes. Staining of each rod with anti-NDRG1a-1 antiserum may not be obvious in Fig. 1a and in Supplementary Fig. S2a, but immunostaining on isolated rods similar to that shown in the right-most panel in Fig. 1b revealed that 100% of rods were immunopositive to NDRG1a-1 in 100 rods examined. Although the reactivity was rather difficult to detect at the nucleus region in rods in Fig. 1a, staining with antiserum against the C-terminal region of NDRG1a-1 with anti-NDRG1a# antiserum gave clear immunopositive signals at the rod nucleus region (vertical white bars in Supplementary Fig. S2c). (Anti-NDRG1a# also recognizes NDRG1a-2, but localization of NDRG1a-2 is different from that of NDRG1a-1, see below). In cones, the entire cell membranes (green signals in Fig. 1a) including those of OS seemed to be NDRG1a-1 immunopositive (lower panels in Fig. 1b). Immunopositive signals were observed at the basal part of cone OS, which could be due to the staining of the calycal processes. Positive signals were also seen at the apical part of the OS, and it appears that NDRG1a-1 is expressed in the whole OS. NDRG1a-1 immunopositive signals seem to be stronger in cones than in rods when signal intensities were compared at the rod nucleus layer (vertical white bars in Supplementary Fig. S2c) and at the cone nucleus layer (the layer above the vertical white bars in Supplementary Fig. S2c).

NDRG1a-2 is expressed only in cones and localized to thin process of cones (green signals in Fig. 1c,d), although the signal itself (arrows in Supplementary Fig. S2b) is not so strong compared with the background. NDRG1b is expressed only in cones and in all four types of cones (green signals in Fig. 1e,f), but not in the rod ellipsoid region (arrowheads in Fig. 1e) or rod nucleus region (Supplementary Fig. S2d) in agreement with our previous study in carp^3^. NDRG1b immunopositive signals were observed in the inner segment, the basal part of the OS (possibly the calycal processes) and the OS in cones (Fig. 1f). In addition to cones, some cells in the inner nuclear layer were also NDRG1b immunopositive (arrows in Supplementary Fig. S2d). Figure 1g shows the schematic representation of localization of each NDRG1 family protein in rods and cones in adult zebrafish.

### Expression of NDRG1 family mRNAs and proteins at early developmental stages

**Figure 2:**
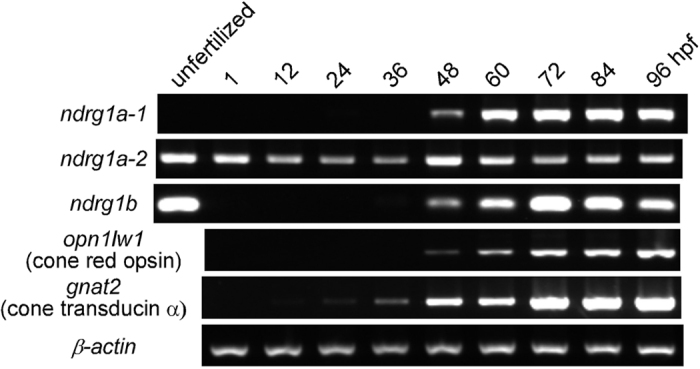
Temporal mRNA expression patterns of NDRG1 family proteins at early developmental stages. Each result shown is from one of two independent studies that gave similar expression patterns. *hpf*, hours postfertilization; *opn1lw1*, red-sensitive cone opsin; *gnat2*, cone transducin α-subunit.

Temporal expression patterns of *ndrg1a-1*, *ndrg1a-2* and *ndrg1b* mRNAs at early developmental stages were examined using total RNAs derived from whole body (Fig. 2). *De novo* mRNA expression of *ndrg1a-1* gene and that of *ndrg1b* gene were greatly increased around 48 hpf, which is correlated well with the expression of mRNAs of cone red-sensitive opsin (*opn1lw1*) and cone transducin α-subunit (*gnat2*), markers of the development of cone photoreceptors, with one notable exception that *ndrg1b* is also expressed in unfertilized eggs. The reason for the disappearance of *ndrg1b* just after fertilization was not known. On the other hand, expression pattern of *ndrg1a-2* remarkably differed from the other two genes. Namely, comparable level of *ndrg1a-2* expression was observed throughout the stages examined and also in unfertilized eggs. In a previous study, *ndrg1a (nbdrg1a-1* and/or *ndrg1a-2*, see above) mRNA expression was observed throughout the early developmental stages (10 hpf-60 hpf) in many tissues^11^. Our result, therefore, suggests that *ndrg1a* mRNA detected in the previous study would be mainly *ndrg1a-2* mRNA.

**Figure 3:**
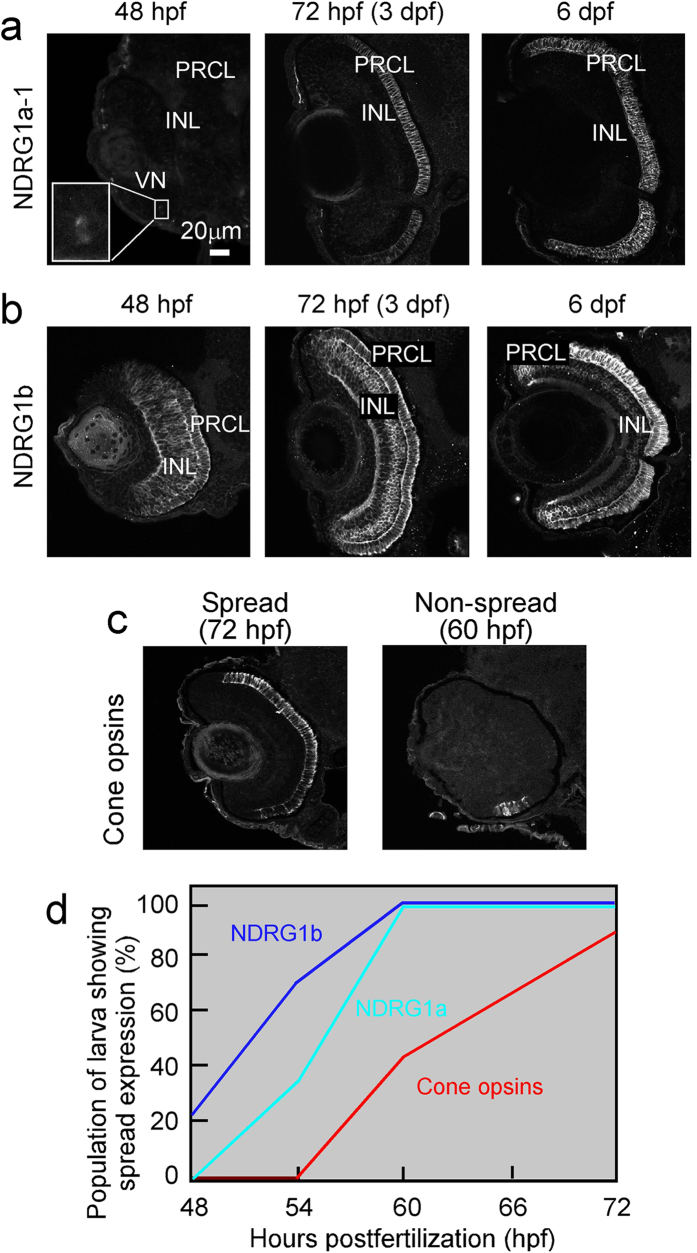
Spatiotemporal expression patterns of NDRG1a-1 and NDRG1b proteins. NDRG1a-1 (**a**) and NDRG1b (**b**) were probed with specific antisera against them at the developmental stages indicated. In (**a**) at 48 hpf, NDRG1a-1 was detected only in the region of ventronasal patch (*VN*), and subsequently it was detected in the photoreceptor cell layer (*PRCL*) but not in the inner nuclear layer (*INL*). In (**b**), NDRG1b was detected at both the PRCL and INL at 48 and 72 hpf, and its distribution was enriched to PRCL at 6 days postfertilization (*dpf*). The expression level of each protein at indicated time was estimated with spread/non-spread criterion (**c**, see Methods), and the result is shown together with that obtained for total cone opsins (**d**). In (**d**), data point is the percentage of the number of the retinas showing “spread” image in the total number of the retinas examined (n = 8–10) at each time point (48, 54, 60 and 72 hpf). Magnifications are the same in (**a**–**c**) (scale bar, 20 μm).

Spatiotemporal expression patterns of these three NDRG1 family proteins in a developing retina were examined using specific anti sera and they were different among the proteins (Fig. 3). Expression of NDRG1a-1 was restricted to the photoreceptor layer (PRCL) at any developmental stages examined (Fig. 3a). It was first detected around 48 hpf in ventronasal patch (VN in Fig. 3a, left) and then throughout the photoreceptor layer at later stages, 72 hpf and 6 dpf (middle and right in Fig. 3a, respectively). Expression of NDRG1b was also detected in the photoreceptor layer around 48 hpf similar to NDRG1a-1, but its expression was also observed in the inner nuclear layer (INL) at this stage (Fig. 3b, left). At later stages, expression of NDRG1b became restricted mainly to the photoreceptor layer (Fig. 3b, right). Detectable signal of NDRG1a-2 was not obtained in the retina at any early developmental stages examined.

Temporal expression pattern of NDRG1 family proteins was compared with that of a total cone opsins including red/green-, blue- and UV-sensitive pigment. Based on a spread/non-spread criteria (Fig. 3c, see Methods), it was found that the onset of expression of NDRG1a and NDRG1b was earlier than that of cone opsins (Fig. 3d).

### Effects of forced-expression of NDRG1 family proteins in rods

In the course of the studies on possible roles of NDRG1 family proteins in photoreceptors, we first knocked down these family proteins with morpholinos because of their convenient usage. Our results suggested that these proteins are related to the formation of OS in both rods and cones. One of these studies is shown in Supplementary Fig. S3 where we used a morpholino against *ndrg1a-1*. In this morphant, OS volume seemed to be reduced without affecting the pigment concentration in both rods and cones. In addition, similar phenotype was observed for morpholinos against *ndrg1b*. (Although *ndrg1b* is specifically expressed in cones, altered cone genesis probably affected rod development which takes place later than cone development^12^).

However, in the study with morpholinos, potentially there could be off-target effects^13^. Therefore, to understand the phenotype of the genetically modified animals more straightforwardly, NDRG1 family proteins were overexpressed (NDRG1a-1) or ectopically expressed (NDRG1a-2 and NDRG1b) in rods in transgenic zebrafish, and morphological and immunohistochemical analyses were made at adult stages. In the followings, we abbreviate the transgenic fish as NDRG1a-1-overexpressing fish (NDRG1a-1-Oe), NDRG1a-2-ectopically-expressing fish (NDRG1a-2-Ee) and NDRG1b-ectopically-expressing fish (NDRG1b-Ee).

**Figure 4:**
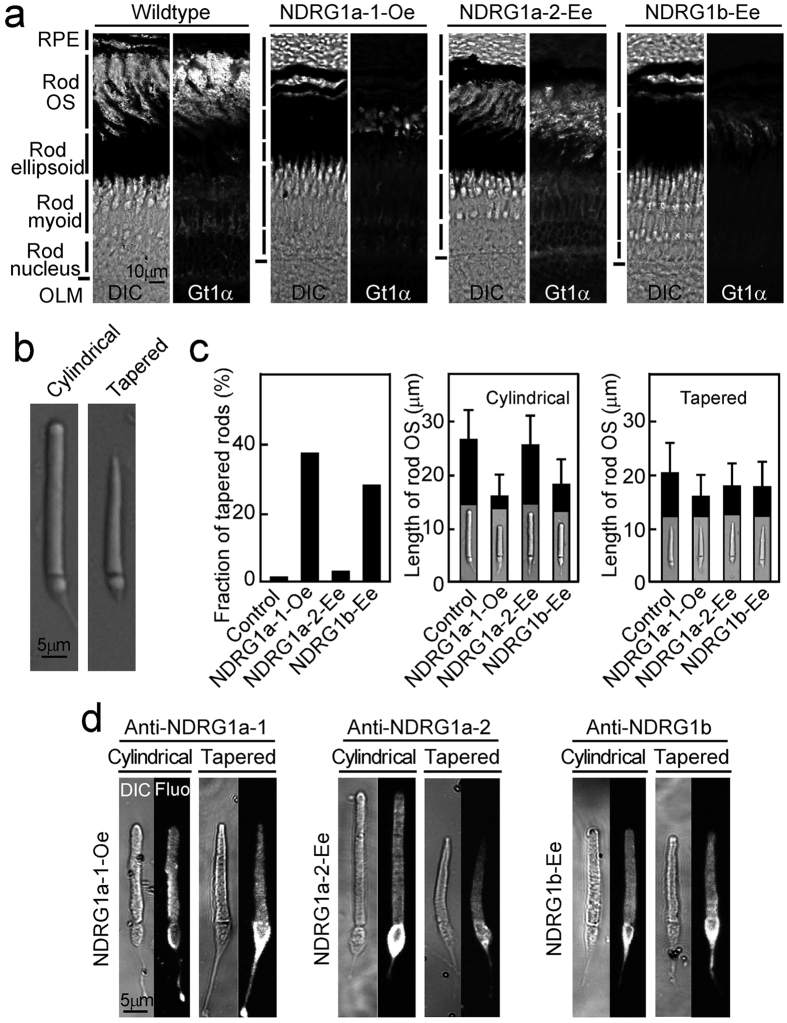
Effects of overexpression of NDRG1a-1, ectopic expression of NDRG1a-2 or NDRG1b in adult zebrafish rods. (**a**) NDRG1a-1 was overexpressed (*NDRG1a-1-Oe*), NDRG1a-2 was ectopically expressed (*NDRG1a-2-Ee*) or NDRG1b was ectopically expressed (*NDRG1b-Ee*) in zebrafish rods. Each adult retina including that of wildtype (*Wildtype*) was sectioned and viewed with differential interference contrast (*DIC*) or immunoprobed with anti-rod transducin α-subunit antibody (*Gt1α*). Magnifications are the same throughout the images in (**a**) (scale bar, 10 μm). Vertical bars from top to bottom in the left of each pair of images show a part of a layer of the retinal pigment epithelium (*RPE*), outer segment, ellipsoid, myoid and nucleus layers, respectively, and horizontal bars show the outer limiting membrane (*OLM*). The rod outer segment layer can be identified with anti-Gt1α-positive signals (right image in each pair). (**b**) A rod with normal cylindrical OS (left) and that with tapered OS (right). Magnifications are the same in (**b**) (scale bar, 5 μm). (**c**) Rods in mCherry-expressing control (*Control*), NDRG1a-1-Oe, NDRG1a-2-Ee or NDRG1b-Ee were isolated, and the fraction of tapered rods was determined (left). The number of rods with tapered OS were 2.0% in Control (217 rods with tapered OS and 10429 rods with cylindrical OS in 5 fish), 38% in NDRG1a-1-Oe (539 tapered and 892 cylindrical in 5 fish), 3.8% in NDRG1a-2-Ee (278 tapered and 7011 cylindrical in 4 fish) and 28% in rods in NDRG1b-Ee (1258 tapered and 3155 cylindrical in 4 fish). The length of the rods showing a cylindrical shape (middle) and the length of the rods showing tapered shape (right) were measured in the population indicated above. Each result shows mean ± SD (middle and right). (**d**) Expression of NDRG1a-1 (left 4 panels), NDRG1a-2 (middle 4 panels) and NDRG1b (right 4 panels) were immunodetected with corresponding specific antiserum in isolated rods showing cylindrical OS (left 2 panels in each set of panels) and those showing tapered OS (right 2 panels in each set) in each transgenic fish. Each rod was viewed with DIC (*DIC)* or immunofluorescently (*Fluo*). Magnifications are the same in (**d**) (scale bar, 5 μm).

Figure 4a shows immunohistochemical studies carried on rod transducin (Gt1α) in transgenic fish. One noticeable point in these studies was that the thickness of the photoreceptor layer, from rod OS to the outer limiting membrane (OLM), was thinner in these transgenic fish than in the wildtype (Fig. 4a). The retinal thickness other than that of the photoreceptor layer and the eye size were not significantly affected in these transgenic fish. The effect was much larger in NDRG1a-1-Oe and NDRG1b-Ee than in NDRG1a-2-Ee. Likewise, rod transducin signal was much reduced in NDRG1a-1-Oe and NDRG1b-Ee.

The above observations prompted us to observe rod OS directly in these transgenic fish. In *Xenopus* retina, it has been shown that at early stages of the development, rod OS shows cone-like shape^14^. In control adult zebrafish expressing mCherry, in addition to the normal rod having a cylindrical OS (Fig. 4b, left), we sometimes observed a rod having tapered OS (Fig. 4b, right) at a very low population (<2%, Fig. 4c, left). In NDRG1a-1-Oe and NDRG1b-Ee, this population increased dramatically (30–40% of total rods), while it did not change significantly in NDRG1a-2-Ee (Fig. 4c, left). Similarly, mean OS length of the normal cylindrical rods was shortened significantly (60–70% of the control) in NDRG1a-1-Oe and NDRG1b-Ee, while in NDRG1a-2-Ee, it was similar to that of control rods (Fig. 4c, middle). The mean OS length of tapered rods was not different significantly among the control and the transgenic fish (Fig. 4c, right). Interestingly, overexpressed or ectopically expressed NDRG1 family proteins were found in the rod OS in addition to the inner segment in all strains no matter whether the OS shape was cylindrical or tapered (Fig. 4d), which is in contrast to the subcellular localization of these proteins in the wildtype rods summarized in Fig. 1g.

### Examination of microscopic membrane structure of tapered rod OS

**Figure 5:**
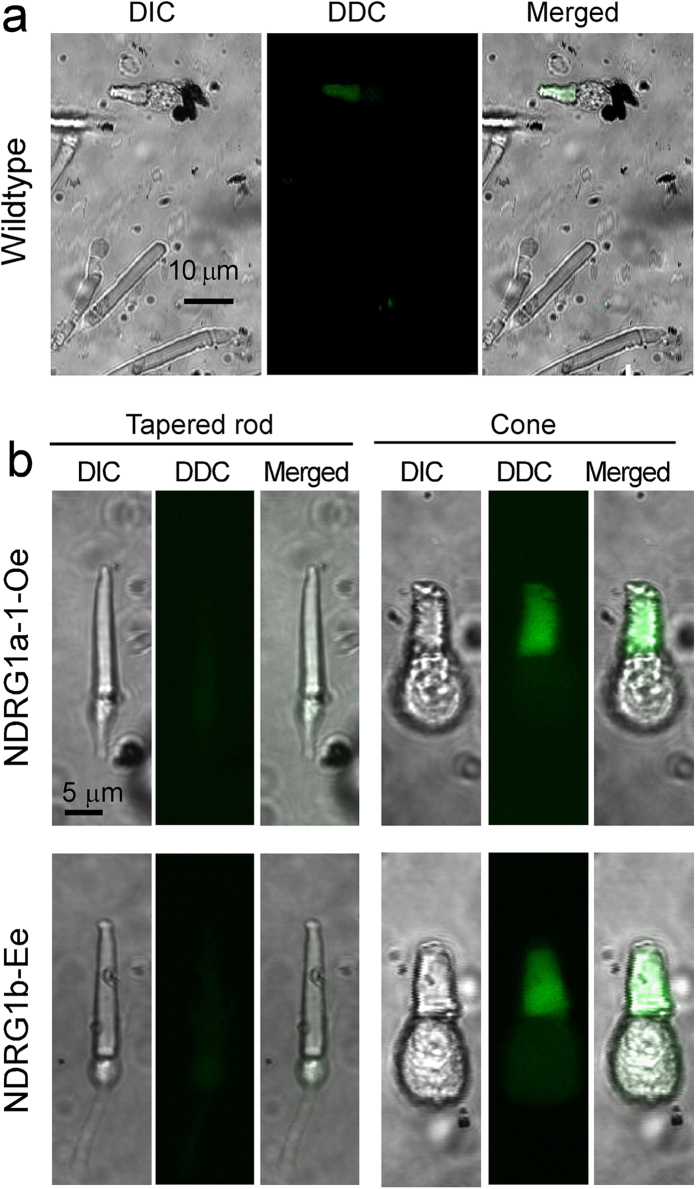
DDC staining of rods showing taper-shaped OS. (**a**) Typical DDC staining of wildtype (*Wildtype*) rods and cones. Only cone OS was stained. (**b**) Rods with tapered OS obtained from NDRG1a-1-Oe (*NDRG1a-1-Oe*) and NDRG1b-Ee (*NDRG1b-Ee*) was negative for DDC staining (upper and lower left 3 panels, respectively). Cone OSs in NDRG1a-1-Oe and NDRG1b-Ee were positively stained (upper and lower right 3 panels, respectively). Cells in (**a**,**b**) were viewed with DIC (*DIC*) and under fluorescent microscope to detect the fluorescence of DDC (*DDC*). Scale bar is 10 μm in (**a**). Magnifications are the same in all of the panels in (**b**) (scale bar, 5 μm).

Macroscopic morphological changes from cylindrical to tapered OS shape observed in rods in NDRG1a-1-Oe and NDRG1b-Ee suggested the possibility that the OS of these rods are made of the infolded plasma membranes similar to the OS of cones. To test this possibility, rods in NDRG1a-1-Oe and NDRG1b-Ee were freshly isolated and stained with *N,N’*-didansyl cystine (DDC), a fluorescent reagent that stains the cone OS surrounded by infolded plasma membranes much more effectively than the rod OS enfolded by cylindrical plasma membrane (Fig. 5a)^15^. Rods with tapered OS in NDRG1a-1-Oe and NDRG1b-Ee did not show fluorescence, but the OS of a cone present in the same visual field was fluorescent (Fig. 5b), which indicated that the rods with tapered OS in either strain do not contain infolded membranes in the OS.

## Discussion

In the present study, we showed that NDRG1 family proteins are expressed in rod and cone photoreceptors in adult zebrafish, although their subcellular localizations are different among the three. NDRG1a-1 is expressed in both rods and cones. However, it is present in rods in the ellipsoid region plus other part except the OS, while in cones it is found in the entire region including the OS (Fig. 1a, b and g). The expression level of NDRG1a-1 seems to be lower in rods than in cones (Fig. 1a and Supplementary Fig. S2a). NDRG1a-2 is found only in the thin process in cones (Fig. 1c, d and g). The amino acid sequence is different between NDRG1a-1 and NDRG1a-2 only at their N-terminal regions (see Results and Supplementary Fig. S1c), and this difference in the sequence should be responsible for this localization difference between NDRG1a-1 and NDRG1a-2. NDRG1b is expressed only in cones, which is consistent with our previous study in carp cones^3^. Its expression was detected in the entire region including the OS (Fig. 1e–g). The difference in the subcellular localization among these family proteins suggests that the role of each of the family proteins is probably slightly different. This notion is supported with the findings that during early development, temporal expression pattern of mRNA (Fig. 2) is different among these family proteins. Spatial expression pattern of the protein is also different. As suggested in this study, NDRG1a-2 seems to be expressed in tissues other than the retina during early development (see Results). NDRG1a-1 expression was restricted to the photoreceptor layer (Fig. 3a) and NDRG1b was first expressed throughout the retina and then its expression was restricted to the photoreceptor layer, most probably to cones (Fig. 3b). Broader spatial expression pattern of NDRG1b together with maternal expression of NDRG1b mRNA (Fig. 2) and early expression of NDRG1b protein (Fig. 3d) suggest more general and important role(s) of NDRG1b than NDRG1a-1 in the retinal development in zebrafish.

Our studies showed that knockdown of NDRG1a-1 or NDRG1b with morpholinos (Supplementary Fig. S3) and overexpression of NDRG1a-1 or ectopic expression of NDRG1b in rods (Fig. 4) all reduced the OS size (see Supplementary Fig. S3 and Fig. 4). These apparently confusing results could be explained by involvement of NDRG1 protein family in cholesterol uptake. It is known that cholesterol content is regulated at different positions in the rod OS^16^: its concentration is high in the rod plasma membrane plus disk membranes at the base of the OS, but it is low at the apical tip. It is possible that the shape of the OS is altered when the content of cholesterol is different from the normal levels. It is known that NDRG1 depletion reduces the content of low-density lipoprotein (LDL) receptor that uptakes free and esterified cholesterol from LDL, and also reduces free cholesterol content at the plasma membrane and the cellular cholesterol content^17^. The length of the rod OS was shortened in an animal model of Smith-Lemli-Opitz syndrome which accompanies reduction of cholesterol levels^18^. Taken together, it is possible that knockdown of NDRG1a-1 or NDRG1b reduced the LDL receptor level and therefore the content of cholesterol to result in the decrease in the OS size (Supplementary Fig. S3). In this case, pigment concentration may not be changed significantly because pigment concentration does not seem to be different at the base and at the tip of a normal rod OS where cholesterol concentration is different. When NDRG1a-1 is overexpressed or NDRG1b is ectopically expressed in rods, possibly LDL receptor expression at the plasma membrane and therefore cholesterol uptake is increased in these rods. Overexpression of NDRDG1a-1 and ectopic expression of NDRG1b in rods both increased the population of tapered OS and shortened OS in rods (Fig. 4). Possible increased cholesterol levels in these rods could cause a change in the lipid composition to alter the shape of the OS from a long cylindrical to a shorter cylindrical or a tapered OS. It is known that LDL receptor is expressed in the photoreceptor inner segment^19^ which is the major site of the endocytosis in photoreceptors^20^. NDRG1a-1 in rods and cones, and NDRG1b in cones are both expressed in the inner segment ellipsoid region where LDL receptor is probably present, which supports the notion that NDRG1a-1 and NDRG1b have some roles in the LDL receptor expression in the ellipsoid plasma membrane.

Based on the above consideration, we tried to examine whether cholesterol level in taper-shaped rod OSs in the transgenic fish is higher than in wildtype rod OSs by using filipin III, a fluorescent polyene macrolide antibiotic that can be used for detection of cholesterol levels^17^. The result was in agreement with our above speculation, and the signal intensity of filipin III was 14–24% higher as a mean in tapered rod OSs than in wildtype rod OSs (124 ± 29% (mean ± SD), n = 18 from three fish, p = 0.00647 in NDRG1a-1-Oe; 114 ± 27%, n = 9 from one fish, p = 0.138, in NDRG1b-Ee). However, signal intensity varied noticeably even in control wildtype rod OSs (roughly ± 50% from the mean, n = 21 from three fish), and we are not fully confident on the values indicated above. For reliable measurements, therefore, we need to use other methods such as quantitative biochemical measurement of cholesterol. Obviously, future studies are needed to know the exact functional mechanisms of the effects we observed in this study.

In Fig. 4, it was shown that overexpressed NDRG1a-1 or ectopically expressed NDRG1b induced the formation of rods with tapered OS. It should be mentioned that ectopically expressed NDRG1a-2 did not show this effect. Because the amino acid sequence of NDRG1a-1 is different from that of NDRG1a-2 only at the N-terminal region, the N-terminal region in NDRG1a-1 is responsible to form tapered rod OS. It is interesting that similar amino acid sequence of the N-terminal region in NDRG1a-1 is present at the N-terminus in NDRG1b (Fig. S1c) which caused similar effects as NDRG1a-1. When NDRG1a-1 was overexpressed or NDRG1b was expressed ectopically, these proteins were present in both the cylindrical and tapered rod OS (Fig. 4d). This result indicates that expression of NDRG1a-1 or NDRG1b in the OS is not associated directly with the OS morphology. This notion is supported with our finding that, so far, rods with tapered OS in the wildtype fish did not show immunoreactivity to NDRG1a-1. Evidently, further studies are required to understand the mechanism of the OS morphological changes induced by the overexpression of NDRG1a-1 and ectopic expression of NDRG1b in rods.

At early developmental stages in *Xenopus*, it is known that rods with “conical” OS but containing disks separated from the plasma membrane are present^14^. In zebrafish, we confirmed that more than 70% of the rod OS shows similar “conical” or short tapered shape in 6 pdf zebrafish. It is evident that the shape of the OS of a rod changes from “conical” to “cylindrical” during development. From the findings that the tapered OS of a rod does not show DDC staining (Fig. 5) and that the shape of the rod OS is conical at early developmental stages, it is evident that the mechanism responsible for the formation of conical or tapered OS is different from the mechanism of the infold of the plasma membrane characteristic to the cone OS. Expression level of NDRG1a-1 seems to be higher in cones than in rods (Fig. 1a), and NDRG1b is additionally expressed in cones. This fact indicates that the expression level of NDRG1 family proteins is much higher in cones than in rods in zebrafish. It has been shown that rods are evolved from cones^21^. It could be the case that during evolution, rods obtained the ability to form a cylindrical OS in the adult by losing NDRG1b and reducing NDRG1a-1 expression levels.

## Methods

For details, see Supplementary Methods.

### Animals

All experiments with zebrafish (*Danio rerio*) in this study were performed in accordance with the institutional guidelines and all experimental protocols were approved by Osaka University Graduate School of Frontier Biosciences (approval number FBS-14-006). *Tupfel long fin* strain was used and the fish were kept under 14 hr light/10 hr dark cycle at 28.5 °C. When necessary, the fish were anesthetized by putting them in ice-chilled water and decapitated during the light period.

### Identification of three NDRG1 family mRNAs

To detect the expression of zebrafish *ndrg1* family mRNAs, coding sequence of each of the known zebrafish mRNAs were used (see Supplementary Methods). Full-length carp (*Cyprinus carpio*) *ndrg1l* (now, *ndrg1b*) cDNA was identified and obtained with a DIG-labeled (Roche) partial coding sequence fragment found previously using a carp retinal cDNA library^3^. Positive clones were sequence-verified.

### Generation of antisera

Antisera against zebrafish NDRG1a-1 and NDRG1a-2 were raised using their partial peptides: Met1-Ala17 in NDRG1a-1 for anti-NDRG1a-1, Met1-Lys30 in NDRG1a-2 for anti-NDRG1a-2, and common C-terminal 54 amino acids in NDRG1a-1 and NDRG1a-2 for anti-NDRG1a# antiserum to detect both NDRG1a-1 and NDRG1a-2 (Supplementary Fig. S1c). To raise anti-NDRG1b antiserum, we used NDRG1b whole protein.

We found polymorphic variants in NDRG1 family proteins in zebrafish (see Results), but amino acid sequences in the partial peptides utilized for generation of anti-NDRG1a-1 and anti-NDRG1a-2 antisera are common in all of the NDRG1a-1 and NDRG1a-2 variants. In the NDRG1b variants, 1 or scattered 6 amino acid substitutions out of 359 total amino acids were present in the variants compared with the amino acid sequence in the NDRG1b protein (NDRG1b_1) used for generation of anti-NDRG1b antiserum. We believe that these substitutions do not affect significantly the activity of anti-NDRG1b antiserum to recognize this protein.

To detect specific binding of each raised antiserum to a zebrafish NDRG1 family protein in immunoblot analysis, we obtained recombinant whole NDRG1a-1 (LC093848.1), NDRG1a-2 (LC093852.1) and NDRG1b (LC093857.1) proteins, with all N-terminally fused with Maltose-binding protein (abbreviated as MBP-NDRG1a-1, for example).

In our preliminary survey, anti-NDRG1a-1 antiserum reacted to MBP-NDRG1b, and anti-NDRG1b antiserum reacted to recombinant NDRG1a-2. To obtain specific antiserum against NDRG1a-1, therefore, anti-NDRG1a-1 antiserum was adsorbed by MBP-NDRG1b. To obtain specific anti-NDRG1b antiserum, anti-NDRG1b antiserum was adsorbed by recombinant NDRG1a-2. Anti-NDRG1a-2 antiserum specifically reacted to MBP-NDRG1a-2.

### Immunohistochemistry and immunocytochemistry

Retinas were immunostained basically according to the method described previously^22^. Light-adapted zebrafish adult and larval eyes were used. When larval eyes were used, they were examined at 48 hours postfertilization (hpf), 54 hpf, 60 hpf and 72 hpf. Immunostaining was also made in isolated rods or cones, and it was performed basically according to the method reported previously^23^. In addition to the antisera raised as above, we used anti-rhodopsin antiserum, and anti-red and green (red/green) opsin antibody reacting to both red- and green-sensitive opsins^22^ to identify the rod and the cone OS, respectively. When temporal cone opsin expression was examined (Fig. 3c), a mixture of anti-red/green-sensitive opsin antibody, anti-blue-sensitive opsin antiserum and anti-UV-sensitive opsin antibody^22^ was used. In addition, to observe the cell morphology of a rod, anti-rod transducin antibody (sc-389; Santa Cruz Biotechnology) was used. To observe the cone morphology, we used a mixture of anti-cone arrestin 1 (cArr1) antiserum and anti-cone arrestin 2 (cArr2) antiserum^22^. Anti-Tom20 antibody (sc-11415; Santa Cruz Biotechnology) was used to identify mitochondria.

### Analysis of temporal expression patterns of NDRG1 family genes and proteins

For temporal analysis of the expression of mRNA of a gene, forty zebrafish larvae each were collected at ~12 hour intervals from 1 hpf to 96 hpf. We also used forty unfertilized eggs. Total RNA was isolated, and 1 μg of it was reverse-transcribed. The cDNAs obtained were used for PCR.

Expression of a protein during development was quantified using spread and non-spread criteria (Fig. 3d). When a protein is expressed in a layer or layers throughout the retina continuously, the expression of that protein is regarded as “spread”. When a protein is expressed in a small area in a layer or layers, or expressed intermittently with gaps horizontally, the expression was regarded as “non-spread”. “Spread expression” was defined by the ratio of the number of retinas showing “spread” to the total number of retinas examined.

### Generation of transgenic zebrafish

In some of the studies, mCherry was expressed in cones in the prenylated and membrane-bound form. The construct, mCherry-HrasCAAX, was produced by the Tol2kit^24^, and ligated to the immediate downstream of the cone specific transducin α subunit promotor^25^.

NDRG1 family proteins were overexpressed (NDRG1a-1) or ectopically expressed (NDRG1b) in zebrafish rods using rhodopsin promoter^26^. As a control, zebrafish expressing unmodified mCherry in rods was produced. In each of these transgenic lines, membrane-bound form of EGFP (mSEGFP) having CAAX prenylation sequence at its C-terminus was also expressed to facilitate the detection of the expression of an NDRG1a family protein.

### Dissociation of rods and cones

Adult zebrafish were dark-adapted overnight, anesthetized in ice-chilled water and decapitated in the dark. Then, both eyes were detached and the retinas were enucleated under a stereomicroscope in dim light. Retinas were treated with Ringer’s solution (119.9 mM NaCl, 2.6 mM KCl, 0.5 mM CaCl_2_, 0.5 mM MgCl_2_, 0.5 mM MgSO_4_, 1 mM NaHCO_3_, 16 mM glucose, 0.5 mM NaH_2_PO_4_, 4 mM HEPES, pH 7.5) containing 1 mg/mL hyaluronidase (Sigma-Aldrich) to obtain isolated rods and cones^27^.

## Additional Information

**How to cite this article**: Takita, S. *et al.* Effects of NDRG1 family proteins on photoreceptor outer segment morphology in zebrafish. *Sci. Rep.* **6**, 36590; doi: 10.1038/srep36590 (2016).

**Publisher’s note:** Springer Nature remains neutral with regard to jurisdictional claims in published maps and institutional affiliations.

## Supplementary Material

Supplementary Information
