# Supplementary material for: Effects of NDRG1 family proteins on photoreceptor outer segment morphology in zebrafish
Source: Sci Rep. 2016 Nov 4;6:36590. doi: 10.1038/srep36590 (PMC5095670; doi:10.1038/srep36590)
Supplement: Supplementary Information [file srep36590-s1.pdf]

Effects of NDRG1 family proteins on photoreceptor outer segment morphology in zebrafish

Shimpei Takita[1], Yasutaka Wada[1,2] and Satoru Kawamura[1,2,]*
[1]Department of Biological Sciences, Graduate School of Science and [2]Graduate School of Frontier Biosciences, Osaka University, Yamada-oka 1-3, Suita, Osaka, 565-0871, JAPAN

Supplementary Information

Supplementary Methods

Identification of three NDRG1 family mRNAs

To detect the expression of zebrafish *ndrg1* family mRNAs, coding sequence of each of the known zebrafish orthologs (NM_001128353.1 for *ndrg1a-1*, NM_213348.3 for *ndrg1a-2*, and NM_200692.2 for *ndrg1b*) was amplified by reverse transcription (RT)-PCR. First-strand cDNA was synthesized from zebrafish adult eye total RNA by reverse transcription using SuperScript III reverse transcriptase (Invitrogen) with an oligo(dT)18 primer. Primer sets used for PCR to obtain full-length NDRG1a family genes are listed in Supplementary Table S1. The PCR products were cloned into pGEM-T-Easy vector (Promega) and amplified. The inserts were verified by DNA sequencing. In our study, there were many single nucleotide polymorphisms in the zebrafish orthologs. We used a representative for each of NDRG1 family proteins (see text): NDRG1a-1_1 (LC093848.1), NDRG1a-2_1 (LC093852.1) and NDRG1b_1 (LC093857.1). Compared with these representatives, in the 6 NDRG1a-1 variants (LC093846.1 - LC093851.1), 1 or 2 amino acid substitutions were found in 3 variants, while in all of 4 NDRG1a-2 variants (LC093852.1 - LC093855.1), amino acid sequences were the same. In the 3 NDRG1b variants (LC093856.1, LC093857.1 and LC102484.1), one variant showed one amino acid substitution, and the other showed 6 substitutions at scattered positions.

Generation of antisera

Partial peptides or a whole protein (Fig. S1c) was used to raise antisera. To raise the antiserum against NDRG1a-1 protein and that against NDRG1a-2 protein, we prepared two partial peptides of NDRG1a-1 (Met1-Ala17) and NDRG1a-2 (Met1-Lys30), respectively. NDRG1a-1 and NDRG1a-2 are different only in these sequences. We also prepared a peptide of a common sequence in both NDRG1a-1 and NDRG1a-2 at their C-terminal 54 amino acids. These peptides were expressed as N-terminally GST-fused ones in *E. coli* BL21 (DE3). For the expression of these peptides, a DNA sequence corresponding to each peptide was PCR-amplified with a primer set containing EcoRI site in the forward primer and SalI site in the reverse primer, subcloned into a pGEM-T-Easy vector and amplified in *E. coli* XL1 Blue. After the sequence was verified, the vector was cut with EcoRI and SalI. Then the insert was ligated to the corresponding sites of pGEX-5X-1 (GE Healthcare) to obtain an N-terminally GST-fused product. Each plasmid was transformed

into *E. coli* BL21 (DE3) for expression. To raise antiserum against NDRG1b protein, we prepared N-terminally GST-fused NDRG1b whole protein similarly as the above peptides. In case when a DNA used for expression of a partial peptide or NDRG1b protein contains a restriction site for NcoI, XhoI or SalI, we replaced the corresponding triplet with that coding the same amino acid but consisting of a different set of a triplet (silence mutation) to obtain a proper product.

A suspension of *E. coli* expressing the GST-fused partial peptides or a whole protein was sonicated, and the supernatant was collected and purified according to the manufacturer's instruction. A peptide or a protein was mixed with Freund's Adjuvant (Sigma-Aldrich) and about 100-200 µg of each peptide or a protein was used to immunize ddY mice.

To examine specificity of each of the antisera, we obtained each of whole proteins of NDRG1 family proteins (LC093848.1 as NDRG1a-1, LC093852.1 as NDRG1a-2 and LC093857.1 as NDRG1b) that were fused with Maltose-binding protein (MBP) at their N-termini (abbreviated as MBP-NDRG1a-1, for example). For this, each of cDNA of NDRG1 family proteins was ligated to EcoRI/SalI sites of pMAL-C2E (New England Biolabs). As a control, we also obtained MBP by introducing a stop codon immediate downstream of DDDDK, a linker to the protein to be expressed. All of the proteins were expressed in *E. coli* BL21 (DE3) and purified according to the manufacturer's protocol. These proteins were then subjected to size-fractionation and the largest bands were collected and stored at -80°C until use.

Crude anti-NDRG1a-1 antiserum and crude anti-NDRG1b antiserum were reacted to NDRG1b and NDRG1a-2, respectively, so that they were purified with adsorption by MBP-NDRG1b or NDRG1a-2. In the purification of anti-NDRG1b antiserum, we used NDRG1a-2 whole protein instead of MBP-fused NDRG1a-2. NDRG1a-2 was prepared by amplifying the cDNA by PCR containing NcoI and XhoI sites, inserted into the corresponding sites in pET-16b (Novagen). Subsequently this protein was expressed in *E. coli* BL21 (DE3). After sonication and centrifugation (15,000 rpm, 4°C, 10 min) the supernatant was used for the source of NDRG1a-2. Anti-NDRG1a-2 antiserum was used without purification.

Selectivity of each of antiserum used was examined with immunoblot as shown in Supplementary Fig. 1d. In addition to this, we also confirmed the selectivity of anti-NDRG1a-1 antiserum and that of anti-NDRG1b antiserum immunohistochemically by checking the selective reduction of the corresponding protein expression level in fish injected with morpholino(s) against NDRG1a-1 and NDRG1b, respectively. Selectivity of NDRG1a-2 antiserum was also confirmed from the fact that it did not show the signal in wildtype rods (Fig. 1c and d, and Supplementary Fig. S2b) but did show it in rods where NDRG1a-2 was forced to be expressed (Fig. 4d).

To examine the expression of NDRG1 family proteins with immunoblot in the zebrafish retina, typically ~20 sheets of adult zebrafish retinas were collected. The fish had been dark-adapted overnight, anesthetized and decapitated in ice-chilled water in the dark. Then, both eyes were detached and retinas were enucleated under a stereomicroscope in dim light. The retinas were solubilized by homogenization in sample buffer (50 mM Tris-HCl, pH 6.8, 2% [w/v] SDS, 10% [w/v] glycerol, 0.0025% bromophenol blue, 1.25% [v/v] ^{2}-mercaptoethanol), heated for 10 mins at 95°C, chilled on ice, snap-frozen and stored at -80°C until use.

Immunoblot analysis was made as described previously[1].

Immunohistochemistry and immunocytochemistry

Expression of NDRG1 family proteins in the retina and their subcellular localization were examined basically according to the method reported previously[2]. Light-adapted zebrafish adult eyes were fixed with a solution containing 4% paraformaldehyde in phosphate-buffered saline (PBS; 10 mM Na_2HPO_4, 1.8 mM KH_2PO_4, 140 mM NaCl, 2.7 mM KCl, pH 7.3) for an hour at 4°C. Then the solution was replaced sequentially with PBS containing 10% sucrose (10% sucrose/PBS), 20% sucrose/PBS for at least 2 hrs each and subsequently 30% sucrose/PBS overnight. Finally the tissues were immersed and embedded overnight in a mixture of OCT compound (Tissue tech) and 30% sucrose/PBS at a 1:1 ratio, and stored at -80°C until use. For larvae, eyes were prepared in the same way as adult eyes except that larval whole bodies at 48 hours postfertilization (hpf), 54 hpf, 60 hpf and 72 hpf were fixed for 13.5 hrs, while at 96 hpf and 6 days postfertilization (dpf) they were fixed for 18 hrs and 24 hrs, respectively.

The fixed eyes cryosectioned at 10 μm thickness were placed on a MAS-coated slide (Matsunami, Osaka, Japan) and dried for 2.5 hrs. After washing out the resin with PBS containing 0.5% Triton X-100 (PBST) three times, the sections were treated overnight at 4°C with a blocking reagent (5% normal goat serum in PBST). After the sections were washed with PBST three times, the samples were incubated overnight at 4°C with antiserum we prepared and when necessary, with antibody commercially available, in the blocking reagent.

Primary antisera and antibodies used in this study were as follows: specific anti-NDRG1a-1 antiserum (1:250 dilution), anti-NDRG1a-2 antiserum (1:200) and anti-NDRG1b antiserum (1:500); anti-Gt1± (1:500) and anti-Tom20 (1:500) antibodies from Santa Cruz Biotechnology (sc-389 and sc-11415, respectively); anti-rhodopsin antiserum (1:2000), anti-red/green opsin antibody reacting to both red- and green-sensitive opsins (1:500), anti-blue opsin antiserum (1:500), anti-UV opsin antibody (1:300), a mixture of anti-cArr1 (cone arrestin 1) antiserum (1:200) and anti-cArr2 (cone arrestin 2) antiserum (1:200) as described previously[2].To detect immunoreactivities, Alexa Fluor 488 anti-mouse, Alexa Fluor 488 anti-rabbit and Alexa Fluor 568 anti-mouse antibodies (Invitrogen) were used at a dilution of 1:500.

Expression of each NDRG1 protein in single isolated cells was examined basically according to the method reported previously[3]. Rods and cones isolated from the retina (see Methods) were attached to a glass slide by centrifugation (300 rpm, 2 min; Cytopro, ELITech). The cells were fixed with 100% methanol for 2.5 min at room temperature and treated for another 1 min with Ringer's solution (119.9 mM NaCl, 2.6 mM KCl, 0.5 mM $CaCl_2$, 0.5 mM $MgCl_2$, 0.5 mM $MgSO_4$, 1 mM $NaHCO_3$, 16 mM glucose, 0.5 mM NaH_2PO_4, 4 mM HEPES, pH 7.5) containing 0.005% Triton X-100 and 5% normal goat serum for permeabilization and blocking, respectively. Other procedures were the same as the immunostaining of the retina in the eye as stated above except that Ringer's solution was used instead of PBST.

The eye sections or isolated cells were observed using an inverted confocal microscope (LSM510META, Zeiss) with plan neofluar 20x/NA 0.5 and C-APO 40x/NA 1.2 water immersion objective lenses.

Analysis of temporal expression patterns of NDRG1 family genes and proteins

Forty zebrafish larvae were collected each time at ~12 hour intervals from 1 hpf to 96 hpf, snap-frozen in liquid nitrogen and stored at -80°C. Frozen larvae were homogenized with 250 µL of TRI Reagent (Sigma-Aldrich) and total RNA was isolated according to the manufacture's instruction. Unfertilized eggs were also used. A total of 1 µg of RNA was primed with an oligo(dT)18 primer and reverse-transcribed with SuperScript III RTase (Thermo Fisher Scientific) or water as a negative control at 42°C for an hour and cDNAs were stored at -80°C until use. RT-PCR was performed using 30 ng of cDNA templates at 94°C for 30 sec, then at 58°C for 30 sec and finally at 72°C for 40 sec. The reactions were repeated for 35 cycles. Primers used for the RT-PCR were listed in Supplementary Table S2.

Morpholino-mediated knockdown of NDRG1a-1 and NDRG1b at early developmental stages

A morpholino against NDRG1a-1 (MO_{1a-1}; 5'-CCATATCGGAGTCTTCCAGAACCAT-3') was synthesized by Gene Tools (Philomath, OR). One- to two-cell-stage zebrafish embryos were injected with 3 ng of this morpholino to inhibit the translation of NDRG1a-1. (Knockdown does not always mean that the expression of the corresponding gene is inhibited by 100 %. It is difficult to determine the degree of inhibition individually because the effects of morpholinos are transient and different individually.) A standard control oligo (Gene tools) was used as a negative control. Microinjection of morpholinos was performed using a glass micropipette made from a glass capillary (G-100; Narishige, Tokyo, Japan) with a pipette puller (P-97 IVF; Sutter, Novato, CA), and by giving N_2 pressure with an electric microinjector (custom-designed IM-30; Narishige). Morpholino-injected larvae were raised in Raising Medium consisting of 0.03% REI-SEA marine salt (Iwaki, Tokyo, Japan) and 106.6 µg/L methylene blue. At 96 hpf, they were collected for phenotypic analysis.

Generation of transgenic zebrafish

Silence mutations were introduced by site-directed mutagenesis prior to construction of an expression vector if each gene had NcoI, XhoI, NotI and/or SalI restriction site in its coding sequence. To identify cones readily, mCherry was expressed in cones in the membrane-bound and prenylated form in some of the studies. The construct, mCherry-HrasCAAX, where CAAX is a prenylation sequence, was produced by the Tol2kit[4]. First, mCherry sequence was amplified from pmCherry-N1 (Clontech) by the primer sets in which HrasCAAX sequence was contained in the reverse primer and mCherry-HrasCAAX was subcloned into pGEM-T-Easy vector to amplify. The insert was excised with NcoI and NotI, and ligated to the downstream of a vector containing a promotor region of cone specific transducin α subunit (T±CP)[5] .

NDRG1 family proteins were overexpressed (NDRG1a-1) or ectopically expressed (NDRG1a-2 and NDRG1b) in zebrafish rods. As a control, zebrafish expressing unmodified mCherry in rods was produced. To establish these transgenic lines, coding sequence of each protein was amplified by PCR with the primer sets containing NcoI site in a forward primer and XhoI site in a reverse primer, and subcloned into pGEM-T-Easy vector to amplify. The inserts were then excised with NcoI and NotI, and ligated to the downstream next to the

rhodopsin promoter in a pCR2.1-TOPO vector (Invitrogen)[6]. Based on a finding by Kwan et al[4], we generated a construct of rhodopsin promoter-NDRG1 family protein (or mCherry in a control)-IRES-mSEGFP-KrasCAAX-6x SV40 late polyA, where IRES stands for slightly modified internal ribosome entry site (Clontech), and mSEGFP, monomeric Super Enhanced GFP[7]. The resultant vectors were introduced into *E. coli* DH5± (DE3) to amplify, and they were purified by QIAGEN Plasmid Midi Kit (QIAGEN). The plasmids were digested and linearized by SalI, purified by phenol-chloroform extraction, precipitated by ethanol, and dissolved in distilled water. The concentration of linearized plasmids was quantified spectrophotometrically and stored at -20º C until use.

The SalI-linearlized plasmid (20 ng/µL) in 2-3 nL of 0.15 M KCl solution containing 0.05% phenol red was injected into the one-cell stage of zebrafish egg. Zebrafish were raised in Raising Medium supplemented with 200 µM 1-phenyl-2-thiourea (nacalai, Kyoto, Japan). At 3 dpf, larvae in which many proportions of rods are positive for mSEGFP were selected with a wide-field epifluorescence microscope (BX51, Olympus) and raised to adult. These adult F_0 fish were crossed with wildtype zebrafish and eggs were raised in Raising Medium containing 1-phenyl-2-thiourea, and those larvae (F_1) carrying the transgene heterologously (+/-) were selected at 3 dpf and further raised until adult. Transgenic zebrafish lines were maintained by selecting these heterologous fish after crossing them with wildtype zebrafish.

Image analysis and statistical analysis

Acquired images shown in Supplementary Fig. S3 were analyzed with ImageJ (Rasband, W.S., ImageJ, National Institutes of Health, Bethesda, MD). Signal intensity of each pixel in the image was converted to an 8-bit representation. Immunopositive area was surrounded with a line manually by eye to determine the size of the area and also the total signal intensity in this area. To determine the concentration of the pigment, the average intensity of an opsin signal was obtained with dividing the total signal intensity by corresponding immunopositive area. Data are presented as mean ± standard deviation, and levels of significance (P value, Student's t-test) are indicated in the figure legends.

***N, N'*-Didansyl cystine staining**

To examine whether rods with tapered OS show the OS similar to that of a cone, we stained the rods and cones with *N,N'*-didansyl cystine (DDC, Sigma-Aldrich) which has been shown to label cone OS much more effectively than rod OS[8]. For this purpose, retinas were dissected and photoreceptors were isolated as described in Methods. A few drops of a suspension of rods and cones were gently mixed with 100 µL of Ringer's solution containing 0.25% (w/v) agarose (nacalai) preincubated at 38°C. A portion of the mixture (~50 µL) was placed on a glass-bottom dish (Matsunami) and then the dish was placed on ice for about a minute to gel the mixture. Then 200 µL of 20 µM DDC dissolved in Ringer's solution was added to the gel for 5 mins at room temperature, and the gel was washed once with Ringer's solution. The dish was filled with Ringer's solution and observed under a confocal microscope (LSM780, Zeiss) with C-APO 40x/NA 1.2 water immersion objective lens. DDC was excited at 405 nm and all the fluorescence longer than 410 nm was

collected.

Supplementary references

1. Tachibanaki, S., Arinobu, D., Shimauchi-Matsukawa, Y., Tsushima, S. & Kawamura, S. Highly effective phosphorylation by G protein-coupled receptor kinase 7 of light-activated visual pigment in cones. *Proc Natl Acad Sci USA*. **102,** 9329-9334 (2005).

2. Tomizuka, J., Tachibanaki, S. & Kawamura, S. Phosphorylation-independent suppression of light-activated visual pigment by arrestin in carp rods and cones. *J. Biol. Chem*. **290**, 9399-9411 (2015).

3. Arinobu, D., Tachibanaki, S. & Kawamura, S. Larger inhibition of visual pigment kinase in cones than in rods. *J. Neurochem*. **115**, 259 –268 (2010).

4. Kwan, K. et al. The Tol2kit: a multisite gateway-based construction kit for Tol2 transposon transgenesis constructs. *Dev. Dyn*. **236**, 3088-3099 (2007).

5. Kennedy, B. N. et al. Identification of a zebrafish cone photoreceptor-specific promoter and genetic rescue of achromatopsia in the nof mutaint. *Invest. Opthalmol. Vis. Sci*. **48**, 522-529 (2007).

6. Asaoka, Y., Mano, H., Kojima, D. & Fukada, Y. Pineal expression-promoting element (PIPE), a cis-acting element, directs pineal-specific gene expression in zebrafish. *Proc. Natl. Acad. Sci. USA* **99**, 15456–15461 (2002).

7. Nomura, M., Nagai, T., Harada, Y. & Tan, T. Facilitated intracellular transport of TrkA by an interaction with nerve growth factor. *Dev Neurobiol*. **71,** 634-649 (2011).

8. Yoshikami, S., Robinson, W. E. & Hagins, W. A. Topology of the outer segment membranes of retinal rods and cones revealed by a fluorescent probe. *Science*. **185**, 1176-1179 (1974).

Supplementary Figures

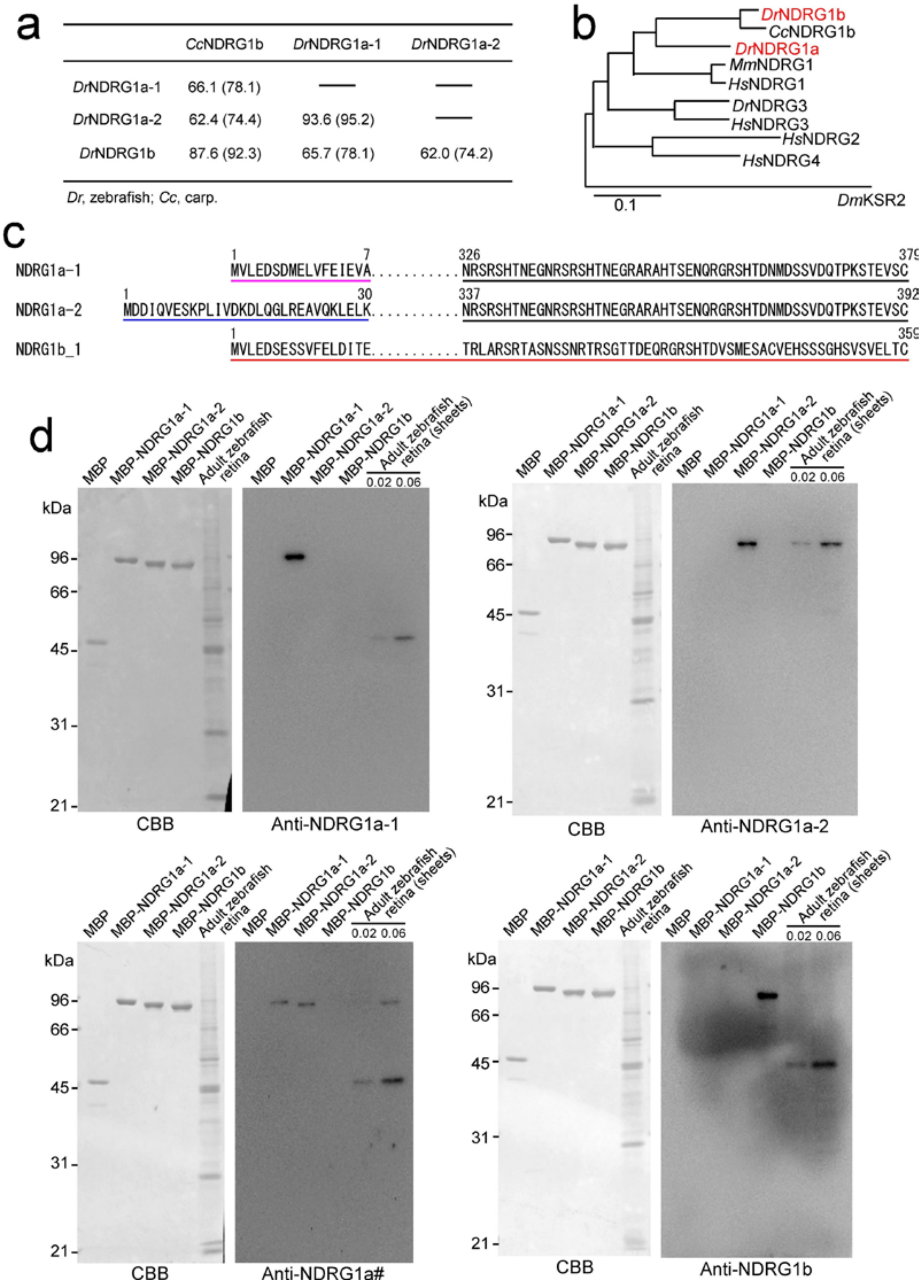

Supplementary Figure S1. Specificity of antiserum raised against NDRG1a-1, NDRG1a-2 or NDRG1b. (**a**) Amino acid identities (similarities in parentheses) among carp NDRG1b (*Cc*NDRG1b, LC102483), zebrafish (*Dr*) NDRG1a-1 (*Dr*NDRG1a-1, NP_001121825.1), *Dr*NDRG1a-2 (NP_998513.2), and *Dr*NDRG1b (NP_956986). (**b**) Phylogenetic tree of NDRG family proteins. *Dr*, zebrafish; *Cc*, carp; *Mm*, mouse; *Hs*, human; *Dm*, fruit fly. Phylogenetic tree was obtained by Neighbor-joining method using the full-length of the identified carp NDRG1b protein sequence (LC102483.1) and NDRG protein sequences in zebrafish (*Dr*NDRG1a-1, NP_001121825.1; *Dr*NDRG1b, NP_956986; *Dr*NDRG3a, NP_955811.1), in human (*Hs*NDRG1 isoform1, NP_006087.2; *Hs*NDRG2 isoform a, NP_963293.1; *Hs*NDRG3 isoform

a, NP_114402.1; *Hs*NDRG4 isoform1, NP_075061) and in mouse (*Mm*NDRG1, NP_032707.2). (**c**) Amino acid sequence used for raising each antiserum (underlined with different colors). There were several polymorphic variants in each of the zebrafish proteins (see text), but for NDRG1a-1 and NDRG1a-2, the amino acid sequences in the regions shown were the same in all of these variants. For NDRG1b, the sequence of one representative (NDRG1b_1) is shown. Note that the N-terminal sequences of several amino acids are similar between NDRG1a-1 and NDRG1b_1 while it is different in NDRG1a-2, and that the rest of the sequence other than Met1-Ala7 in NDRG1a-1 and Met1-Lys30 in NDRG1a-2 is the same between NDRG1a-1 and NDRG1a-2. (**d**) Specificity of each purified antiserum against NDRG1a-1 (upper left), NDRG1a-2 (upper right), NDRG1a-1 plus NDRG1a-2 (lower left), and NDRG1b (lower right). In each pair of the panels, Coomassie Brilliant Blue (*CBB*)-staining (left) and immunoblot staining (right) are shown. Proteins used for detecting specificity were all N-terminally MBP-fused NDRG1 family proteins (see Supplementary Methods). Each NDRG1 family protein was also detected in adult zebrafish retina. The amount of the retina probed is indicated as portions of a retina (*sheets*). In these sheets, single bands corresponding to the calculated molecular masses were detected for NDRG1a-1 (upper left) and NDRG1b (lower right). Anti-NDRG1a-2 recognized a single band of ~90 kDa (upper right), which was also detected with anti-NDRG1a# (lower left). The results suggest that the ~90 kDa band is a homodimer of NDRG1a-2.

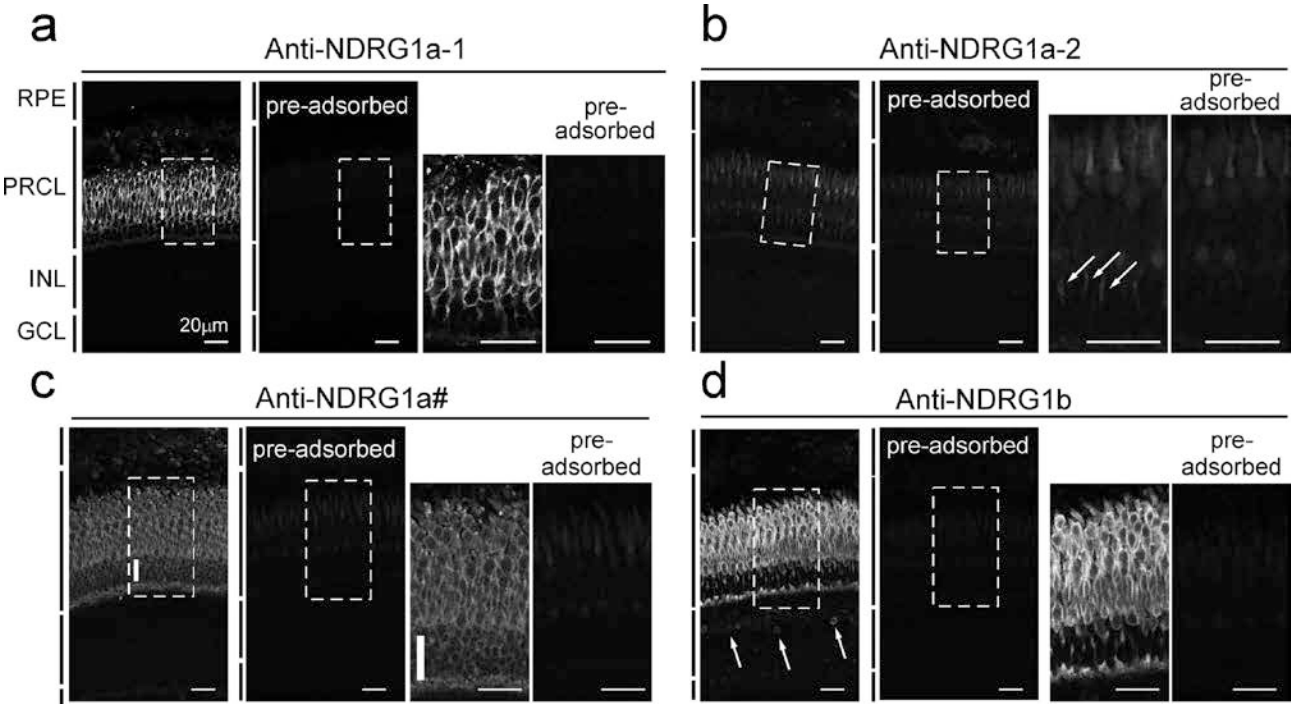

Supplementary Figure S2. Localization of NDRG1a-1, NDRG1a-2 and NDRG1b proteins in zebrafish retina. Retinal sections were immunoprobed with anti-NDRG1a-1, anti-NDRG1a-2, anti-NDRG1a#, or anti-NDRG1b antiserum (leftmost panel in each of **a** - **d**). In (**a**) - (**d**), control measurement was made with each antiserum pre-adsorbed by each corresponding NDRG1a family protein (second left panels in **a** - **d**). The area surrounded by a dotted rectangular was magnified and is shown in the right two panels in (**a**) - (**d**). Arrows in (**b**) indicate the thin cone process detected by anti-NDRG1a-2 antiserum. Thick vertical white bars in (**c**) indicate the immunostaining of NDRG1a-1 in the plasma membranes surrounding the rod nucleus (see text). Arrows in (**d**) indicate NDRG1b positive cells in the inner nuclear layer. *RPE*, retinal pigment epithelium; *PRCL*, photoreceptor cell layer; *INL*, inner nuclear layer; *GCL*, ganglion cell layer. Scale bars indicate 20 μm throughout this figure.

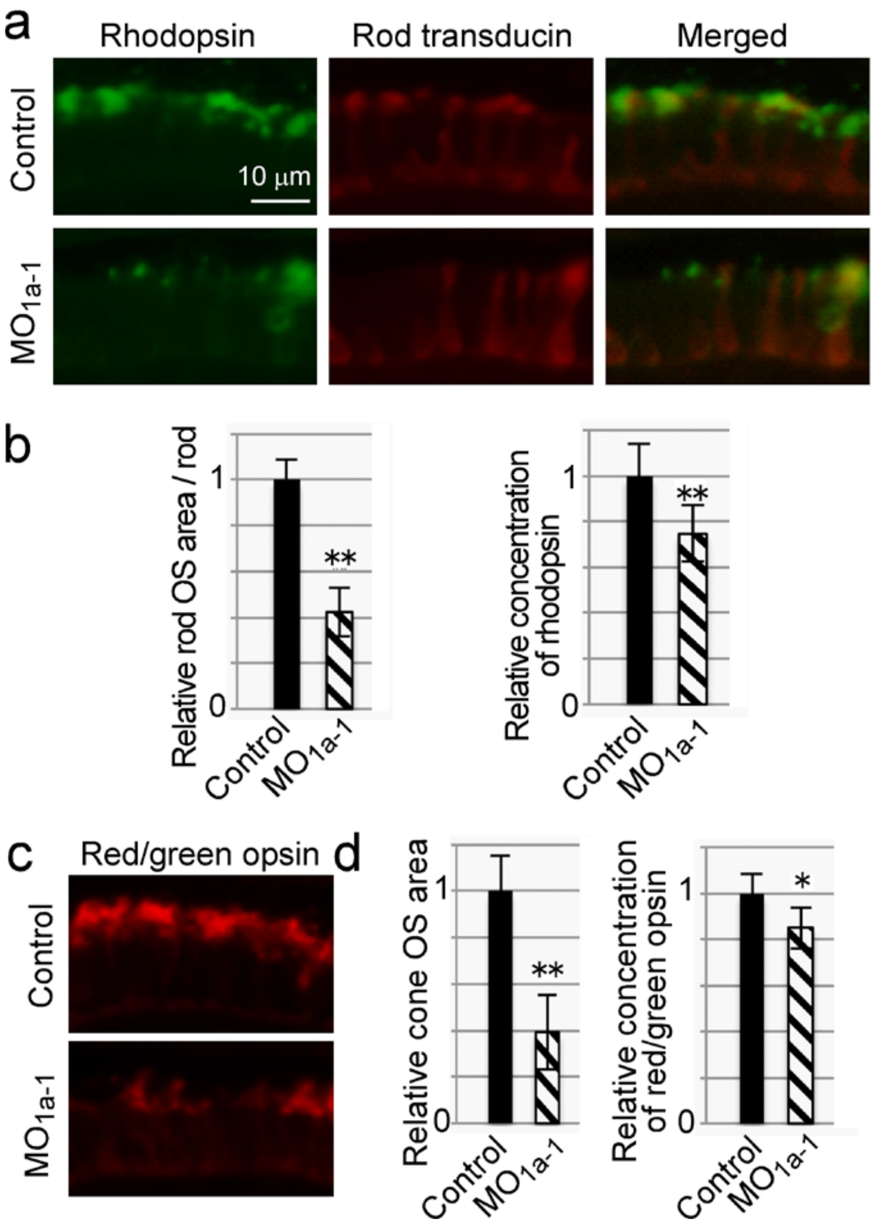

Supplementary Figure S3. Effects of NDRG1a-1 knockdown with a morpholino against ndrg1a-1 (MO_{1a-1}). (**a**) At 96 hpf, effects of MO_{1a-1} on photoreceptors were examined. In the retinas of control morpholino-injected (*Control*) and MO_{1a-1}-injected fish (MO_{1a-1}), expression of rhodopsin and rod transducin were immunodetected. (**b**) From studies as shown in (**a**), rod OS area was determined from rhodopsin-immunopositive region (left panels in **a**) and the number of rods was determined from rod transducin-immunopositive cells (middle panels in **a**). From these determination, relative rod OS area per rod (left) and relative rhodopsin concentration (right) were estimated for the retinas of control and MO_{1a-1}-injected fish (n = 11 for both *Control* and MO_{1a-1}). (**c**) In the retina from MO_{1a-1}-injected fish, red/green-sensitive opsin was immunodetected. (**d**) Relative cone OS area (left) and relative concentration of red/green-sensitive opsin (right) were estimated in the retinas of control and MO_{1a-1}-injected fish as in (**b**) (n = 11 for *Control* and n = 9 for MO_{1a-1}). Magnifications are the same in (**a**) and (**c**) (scale bar, 10 μm). $*P < 0.005$, $**P < 0.0000005$.

Supplementary tables

Supplementary Table S1. Primer sequence for cloning of NDRG1 family proteins.

Primer	Sequence (5'-3')
ndrg1a-1, Forward	GCGCACACAAGAGCTTTACTT
ndrg1a-2, Forward	CACGCGTCCGGTAGTCAATTGTCTTC
ndrg1a-1 and ndrg1a-2, Reverse	CGATGTCTCTGTGCTGCATTGC
ndrg1b, Forward	GCGCCACACTGTAAACAAGAGG
ndrg1b, Reverse	CTCCCCCAGGCATTCATTCACA

Supplementary Table S2. Primer sequence for spatio-temporal expression pattern.

Primer	Sequence (5'-3')
ndrg1a-2, Forward	TGAGGGAGGCTGTCCAGAAA
ndrg1a, Reverse	GGTGACGGTACCTGCCAATG
ndrg1b, Forward	CAGCACACCAGTCTTTTACAGCA
ndrg1b, Reverse	AGGACCATTGGTAGGCTCTCAGA
opn1lw1, Forward	GGGAAGCAATGTTCACATATACC
opn1lw1, Reverse	CATCTTTCCCAAGAGATGACAGT
gnat2, Forward	GAGGATAAGGAAATGGCCAAGAA
gnat2, Reverse	GAGGTTCTGGAGCTTTTGACTA
^{2} *-actin*, Forward	CATCGGCAATGAGCGTTTCCGTT
^{2} *-actin*, Reverse	GTTCGAGAGTTTAGGTTGGTCG
